# Effect of 6-week curcumin supplementation on aerobic capacity, antioxidant status and sirtuin 3 level in middle-aged amateur long-distance runners

**DOI:** 10.1080/13510002.2022.2123882

**Published:** 2022-09-20

**Authors:** Sebastian Bańkowski, Miroslav Petr, Michał Rozpara, Ewa Sadowska-Krępa

**Affiliations:** a Institute of Sport Sciences, the Jerzy Kukuczka Academy of Physical Education, Katowice, Poland; b Department of Physiology, Faculty of Physical Education and Sport, Charles University, Prague, Czech Republic

**Keywords:** flavonoids, Supplementation, antioxidant enzymes, non-enzymatic antioxidants, oxidative stress, prooxidant/antioxidant homeostasis, men, training

## Abstract

**Background:**

The study was undertaken to evaluate the effect of 6-week supplementation with a daily dose of 2g of curcumin on VO_2max_ and prooxidant/antioxidant homeostasis in middle-aged amateur long-distance runners during the preparatory period of the macrocycle.

**Methods:**

Thirty runners were randomly assigned to a placebo group (PL) and a curcumin-supplemented group (CU). Their VO_2max_ was assessed before supplementation and after 6 weeks of supplementation. Venous blood samples were collected from the participants at rest, immediately after exercise, and after 1h of recovery to evaluate the activity of antioxidant enzymes (SOD, CAT, GPx), non-enzymatic antioxidants (GSH, UA) and sirtuin 3 level (SIRT 3), as well as the levels of oxidative stress markers (TOS/TOC, MDA, and 8-OHdG) and muscle damage markers (CK, LDH, and Mb).

**Results:**

VO_2max_, the activity of enzymatic antioxidants, the concentrations of non-enzymatic antioxidants, the levels of oxidative stress markers, and the levels of muscle damage markers did not change significantly in the CU group over 6 weeks of supplementation with curcumin. However, the resting concentration of SIRT 3 was found to be significantly higher (p ≤ 0.05) compared with pre-supplementation.

**Conclusion:**

Curcumin supplementation does not have a significant effect on VO_2max_ and prooxidant/antioxidant homeostasis in runners.

## 1. Introduction

Oxygen uptake during endurance exercise increases more than 10-15-fold compared with resting uptake, which can lead to excessive production of reactive oxygen species (ROS), mainly due to an increased flow of electrons through the respiratory mitochondrial chain. The oxidation of haemoglobin into methaemoglobin, the xanthine oxidase reaction, the autoxidation of catechol-amines, and the NADPH oxidase of phagocytes [1–5] are also reported as the sources of additional production of ROS. Under physiologically normal circumstances, ROS facilitate cellular energy metabolism and signal transduction and help regulate gene expression, but strenuous physical exercise raises their levels which disturbs prooxidant/antioxidant homeostasis towards oxidative reactions [6–8] and creates a risk of oxidative damage dependent on workout intensity and duration [8] or of alterations in cell structures and components [9–11]. However, exercise-induced production of ROS also enables hormesis-like adaptations, including an up-regulation in endogenous antioxidant defences [7,12].

Athletes use various means to attenuate the adverse effects of physical exercise and maximize its benefits [13]. Many endurance athletes take antioxidant supplements that mitigate oxidative stress, shorten the post-exercise recovery-time, and improve aerobic capacity [13]. One of these supplements is curcumin, a polyphenol compound extracted from the root of Curcuma longa L., [14], which involves a very low risk of noticeable side effects and is safe in daily doses of between 2 and 8 g /day. The effectiveness of curcumin as an antioxidant depends on training volume and intensity and its dietary intake [13, 15].

According to research, curcumin induces the transcriptional activation of the nuclear factor erythroid 2-related factor 2 (Nrf2) that mitigates damage caused by ROS [16]. It is also reported to scavenge ROS, prevent the excessive production of lipid peroxides [17–19], enhance the plasma antioxidant capacity [20], and increase the activity of sirtuin 3 (SIRT3) that inhibits the production of ROS [21], and to stimulate the expression of antioxidant enzymes [22–24]. In the existing studies on curcumin, participants have been supplemented with curcumin from 2 days to 12 weeks before performing exercise tests [25]. As with some other supplements that dampen the oxidative and pro-inflammatory response to exercise, more research is needed to determine whether curcumin can increase the training-response signal for adaptation, especially that its effect on the magnitude and efficacy of training response and adaptability in endurance runners is yet not known.

Given the above, this study sought to establish whether 6-week supplementation with a daily dose of 2 g of curcumin would influence the aerobic capacity and prooxidant/antioxidant homeostasis in middle-aged amateur long-distance runners during the preparatory phase of the macrocycle. The hypothesis being tested was that curcumin would enhance the antioxidant effect of training and improve the athletes’ aerobic capacity.

## 2. Methods

### 2.1. Participants

The study participants were 30 middle-aged amateur long-distance runners (aged 38.33 ± 5.28 years), who were recruited on a voluntary basis from sports clubs in the Silesian Voivodship (Poland). At the time of the study, all athletes were in the preparatory phase of the macrocycle.

The inclusion criteria included male gender and running experience of at least 3 years. Athletes who used tobacco, alcohol, or any medicines or dietary supplements in the four weeks preceding the study were excluded from it. All the eligible participants were informed about the purpose and design of the study and submitted written consent to participate in it.

The participants were randomly divided between a placebo group (PL; n = 15) and a group supplemented with curcumin (CU; 2 g/day for six weeks; n = 15). The basic characteristics of both groups are presented in Table 1.

**Table 1.** Basic characteristics of participants.

Variables	PL (n = 15)		CU (n = 15)	
	M (SD)	Min-Max	M (SD)	Min-Max
Age (years)	39.6 (5.8)	31.0-49.0	37.1 (4.5)	31.0-44.0
Height (cm)	177.4 (6.3)	170.0-188.0	178.3 (7.9)	162.0-190.0
Weight (kg)	76.7 (5.5)	68.1-87.0	74.4 (11.1	53.8-91.0
BMI (kg/m^2^)	24.4 (1.9)	20.4-27.4	23.3 (2.1)	20.5-26.6
Internship (years)	4.0 (0.9)	3.0-6.0	4.3 (1.0)	3.0-6.0

Notes: M-mean; SD – standard deviation; PL - placebo group; CU – curcumin - supplemented group

The study was set up in conformity with the ethical guidelines of the World Medical Association Declaration of Helsinki and was approved by the local Bioethics Committee (certificate no. 11/2019). It was registered with the Clinical Trials Registry (ACTRN12622000456752) on 23 March 2022.

### 2.2. Study design

The study was conducted during the preparatory phase of the macrocycle, when the study participants ran an average of 104.37 ± 13.30 km a week (min–max: 85 km/week – 130 km/week) with an average speed of 4.83 ± 0.32 min/km (min–max: 4.22 min/km – 5.02 min/km). Their training plans were based on Daniel’ s running formula [26] and consisted of five training units varying in exercise intensity: 1) easy pace (59%−74% of VO_2max_; 25-30% of the weekly running distance), 2) marathon pace (75%−84% of VO_2max_; 15-20% of the weekly running distance), 3) threshold pace (83%−88% of VO_2max_; not more than 10% of the weekly running distance), 4) interval pace (95-100% of VO_2max_, less than 8% of the weekly running distance), and 5) repetition pace (105-120% of VO_2max_; less than 5% of the weekly running distance).

Maximal oxygen uptake (VO_2max_) was measured before (a first trial) and after 6 weeks of supplementation with curcumin (a second trial) in participants connected to a breath-by-breath gas analyser (MetaLyzer 3B-R2, Leipzig, Germany) and performing an incremental exercise test on a treadmill (Cosmed, Germany). The treadmill speed was increased by 2 km/h every 3 min until the running speed of 14 km/h, after which the treadmill incline was raised in 2.5-degree increments every 3 min and the test continued until voluntary exhaustion. The protocol of the exercise test was created based on studies conducted by other authors [27,28]. Venous blood samples for biochemical analysis were taken from participants’ antecubital veins at rest and after 3 and 60 min of recovery.

### 2.3. Supplements

The shells of capsules containing curcumin or placebo were made of a soft substance consisting of gelatine and purified water (Nanga, Złotów, Poland). One capsule held 500 mg of common turmeric extract standardized to contain 95% curcumin (500 mg) and 95% piperine (10 mg) or corn-starch (400 mg) and riboflavin pigment (10 mg). Participants in both groups were instructed to take two capsules after breakfast and another two after dinner, washing them down with a glass of water.

### 2.4. Biochemical analyses

Part of fresh whole blood samples was immediately assayed for reduced glutathione (GSH) by a colorimetric method with 5.5′-dithiobis-2-nitrobenzoic acid [29], and hematocrit was assayed using a micro-hematocrit method (Hettich 210, DJB Labcare, UK). The remaining blood was placed in the test tubes to separate plasma (BD Vacutainer PPT™ Plasma Preparation Tube, UK) and serum (BD Vacutainer™ Serum Tube, UK). Plasma was obtained by centrifuging the tubes for 10 min at 1000 × g at 4 ˚C (SIGMA 2-16KL, Sigma Laborzentrifugen GmbH, Germany). Erythrocyte sediments thus obtained were washed three times with cold saline (4 °C). To extract serum, the test tubes were allowed to stand for 30 min for blood to clot and then were centrifuged at 1000 × g at 4 ˚C. Blood plasma, serum and erythrocytes were stored less than one month at −80 ˚C before they were assayed.

The activity of the following antioxidant enzymes (superoxide dismutase – SOD, EC 1.15.1.1, glutathione peroxidase – GPx, EC 1.11.1.9 and catalase – CAT, EC 1.11.1.6) was analysed. SOD activity was measured with a commercially available RANSOD 125 kit (Randox, UK), which uses xanthine and xanthine oxidase (XOD) to generate superoxide radicals which react with 2-(4-iodophenyl)−3-(4-nitrophenol)−5-phenyltetrazolium chloride (I.N.T.) to form a red formazan dye. The intra- and inter-assay CV for SOD were 4.11% and 6.51%, respectively.

The activity of GPx was assayed using a commercially available RANSEL RS505 kit (Randox, UK) utilising the method developed by Paglia and Valentine [30]. GPx catalyses the oxidation of GSH by cumene hydroperoxide. In the presence of glutathione reductase (GR) and NADPH, the oxidised glutathione (GSSG) is immediately converted to the reduced form with a concomitant oxidation of NADPH to NADP^+^. The decrease in absorbance at 340 nm is measured. The intra- and inter-assay CV for GPx were 5.83% and 4.03%, respectively.

The activity of CAT was assayed by the Aebi method [31] and expressed at the rate constant (k) of a first order reaction of hydrogen peroxide decomposition related to the haemoglobin (Hb) content (k/gHb).

The activity of all antioxidant enzymes was measured at 37 ˚C and expressed per 1 g of haemoglobin assayed using a standard cyanmethaemoglobin method and a diagnostic kit (HG980, Randox, UK).

The activity levels of creatine kinase (CK, 2.7.3.2) and lactate dehydrogenase (LDH, EC 1.1.1.27) and the concentrations of uric acid (UA) in fresh plasma samples were determined using the Randox Laboratories diagnostic kits (UK; CK522, LD401, and UA230, respectively).

CK activity was measured using creatine phosphate and adenosine-5'-diphosphate (ADP) as substrates. LDH activity was assessed based on the reduction reaction of pyruvate to lactate in the presence of NADH. The concentration of UA was determined using a colorimetric method, where UA is converted by uricase to allantoin and hydrogen peroxide, which under the catalytic influence of peroxidase, oxidizes 3,5-dichloro- 2-hydroxybenzenesulfonic acid and 4-aminophenazone to form a red-violet quinoneimine compound*.* The intra- and inter-assay coefficients of variation (CV) were 1.93% and 3.63% (CK), 2.83% and 3.38% (LDH) and 0.38% and 5.64% (UA).

The concentrations of plasma lipid peroxides were assayed using the thiobarbituric acid (TBA) test following the method by Buege and Aust [32] modified by adding 0.01% butylated hydroxytoluene to lower the metal-catalysed auto-oxidation of lipids during heating with the TBA reagent. The chromogen was extracted using n-butanol and the absorbance of the organic layer was read at 532 nm [33]. Lipid peroxide concentrations were expressed as micromoles of malondialdehyde (MDA) per litre of plasma. MDA was calculated from the calibration curve prepared with 1,1,3,3-tetraethoxypropane.

The serum concentration of total antioxidant status (TAS) was determined using a colorimetric method (the Randox Laboratories Ltd. diagnostic kit, UK) and ABTS® (2,2’- azino-di-[3-ethylbenzthiazoline sulphonate]) incubated with a peroxidase (metmyoglobin) and H_2_O_2_ to produce the radical cation ABTS®^+^, which was measured at 600 nm. The intra- and inter-assay CV for TAS were 1.20% and 1.77%, respectively.

The total oxidant status/total oxidant capacity (TOS/TOC) was measured by a photometric method (the KC5100 PerOx test kit, Immundiagnostik AG, Germany). The intra- and inter-assay CV for TOS/TOC were 2.94% and 6.74%, respectively.

The concentrations of myoglobin (Mb) and sirtuin 3 (SIRT 3) were assessed by a sandwich enzyme immunoassay for in vitro quantitative measurement of Mb and SIRT 3 in human serum (SEA480Hu and SEE913Hu, respectively, Enzyme-linked Immunosorbent Assay Kits, Cloud-Clone Corp. USA). The intra- and inter-assay CV for both markers were < 10% and < 12%.

The measurement of 8-hydroxy-2’-deoxyguanosine (8-OHdG) was performed using a DNA damage kit (K059-H1, Arbor Assays, USA). The intra- and inter-assay CV for 8-OHdG were 7.1% and 8.1%, respectively.

All biochemical tests were conducted by a certified biochemistry laboratory in conformity with the PN-EN ISO 9001:2015 standard and following the recommendations of the tests manufacturers.

### 2.5. Statistical analysis

The data below represent means (M) ± standard deviations (SD), medians (Me), and quartile deviations (QD). The normality of data distributions, homogeneity of variance, and sphericity were assessed using the Shapiro–Wilk test, Levene’s test, and Mauchly's test, respectively. Non-parametric tests were applied when the criteria for normal data distribution, homogeneity of variance, and sphericity were not met. The significance of between-group differences was determined using the Mann–Whitney test. Within-group differences were assessed for significance by Wilcoxon matched-pairs signed-rank test and Friedman rank test followed, when appropriate, by Dunn’s post-hoc test. The level of significance for all tests was α = 0.05. The statistical analysis was performed in IBM Statistics 26.0 (IBM Corporation, Armonk, NY, USA).

## 3. Results

### 3.1. Aerobic capacity

The pre-supplementation levels of VO_2max_ were not significantly different between the supplemented group and the placebo group. Measurements at week 6 showed that they slightly (non-significantly) increased in both groups (from 52.4 ± 5.4 ml.kg^−1^.min^−1^–50.8 ± 4.4 vs. in the PL group and from 52.0 ± 0.7 ml.kg^−1^.min^−1^–49.9 ± 5.3 in the CU group).

### 3.2. Antioxidant status

The results of the biochemical analysis of enzymatic (SOD, CAT, GPx) and non-enzymatic antioxidant defence (GSH, UA) components are summarised in Table 2. The activity of SOD, CAT and GPx in participants supplemented with curcumin for 6 weeks was not significantly different from that recorded at baseline. The post-exercise activity of CAT and GPx determined after the first and second trials did not significantly differentiate the CU group from the PL group. In both trials, GPx activity recorded for the CU group was significantly lower 1 h after exercise than immediately afterward. In the PL group, GPx activity obtained after 1 h of recovery was significantly lower (*p* ≤ 0.001) than immediately after exercise only in the second trial. Also, in the second trial, it was significantly higher immediately after exercise than at rest (*p* ≤ 0.001).

**Table 2.** Changes in markers of antioxidant status and sirtuin 3 level after 6-week curcumin supplementation and exercise.

Variables	Measurement	Time	PL (n = 15)	CU (n = 15)
			Me (QD)	Me (QD)
SOD (U/gHb)	1-st	rest	1634.4 (233.4)	1630.3 (228.1)
		post test	1594.2 (227.6)	1544.5 (171.4)
		1 h post test	1564.4 (157.6)	1736.3 (228.8)
	2-nd	rest	1552.4 (160.9)	1713.3 (222.1)
		post test	1607.2 (160.1)	1670.3 (249.0)
		1 h post test	1652.2 (186.1)	1688.3 (158.2)
CAT (k/gHb)	1-st	rest	169.7 (28.3)	188.3 (12.5)
		post test	190.5 (27.2)	204.7 (41.4)
		1 h post test	184.6 (10.7)	209.9 (27.8)
	2-nd	rest	199.2 (40.2)	178.0 (20.6)
		post test	191.7 (32.6)	204.5 (30.0)
		1 h post test	181.3 (14.3)	170.9 (30.9)
GPx (U/gHb)	1-st	rest	42.8 (6.3)	43.0 (4.7)
		post test	49.3 (6.1)	50.1 (4.2)
		1 h post test	44.8 (8.0)	42.4 (5.3) ^bbb^
	2-nd	rest	46.0 (8.4)	41.2 (5.0)
		post test	53.3 (12.2)	50.4 (12.0) ^ccc^
		1 h post test	43.4 (6.0) ^bb^	44.7 (7.1) ^bb^
GSH (µg/mgHb)	1-st	rest	2.7 (0.3)	2.7 (0.3)
		post test	2.7 (0.3)	2.7 (0.3)
		1 h post test	2.9 (0.5)	3.0 (0.3)
	2-nd	rest	2.7 (0.3)	2.8 (0.2)
		post test	2.7 (0.2)	2.8 (0.2)
		1 h post test	2.8 (0.4)	2.9 (0.3)
UA (mg/dl)	1-st	rest	5.5 (1.0)	4.8 (1.0)
		post test	5.2 (0.5)	4.8 (1.1)
		1 h post test	7.1 (0.9) ^bbbcc^	6.6 (1.2) ^bbbccc^
	2-nd	rest	5.4 (0.6)	4.6 (1.5)
		post test	5.3 (0.5)	4.6 (1.3)
		1 h post test	6.5 (0.8) ^bbbccc^	6.7 (1.5) ^bbbcc^
TAS (mmol/l)	1-st	rest	1.5 (0.1)	1.3 (0.1) ^ddd^
		post test	1.4 (0.1)	1.2 (0.1) ^ddd^
		1 h post test	1.6 (0.1) ^bbbc^	1.4 (0.1) ^bbbddd^
	2-nd	rest	1.4 (0.1)	1.3 (0.2)
		post test	1.3 (0.1) ^a^	1.3 (0.1)
		1 h post test	1.5 (0.1) ^bbb^	1.6 (0.2) ^bbbc^
SIRT3 (ng/ml)	1-st	rest	2.6 (1.0)	2.2 (0.5)
		post test	4.0 (2.4)	3.0 (0.7) ^c^
		1 h post test	3.8 (2.0)	2.7 (1.4)
	2-nd	rest	1.4 (1.2) ^a^	2.9 (1.0) ^ad^
		post test	3.5 (1.7) ^cc^	4.8 (1.7)
		1 h post test	3.3 (1.1) ^cc^	3.3 (2.0)

Notes: Me – median; QD – quartile deviation; PL – placebo group; CU – curcumin – supplemented group; SOD – superoxide dismutase; GPx – glutathione peroxidase; CAT – catalase; GSH – reduced glutathione; UA – uric acid; TAS – total antioxidant status; SIRT 3 – sirtuin 3; ^a^*p *≤ 0.05 – significantly different vs. values in the first trial (Wilcoxon matched-pairs signed rank test); ^bb^*p *≤ 0.01, ^bbb^*p *≤ 0.001 – significantly different *vs.* values immediately post-exercise in the same trial (Friedman rank test and Dunn’s post-hoc test); ^c^*p *≤ 0.05, ^cc^*p *≤ 0.01, ^ccc^*p *≤ 0.001 – significant different *vs*. values at rest in the same trial (Friedman rank test); ^d^*p* ≤ 0.05, ^ddd^*p* ≤ 0.001 – significantly different vs. the corresponding values in the placebo group (Mann-Whitney test)

Neither supplementation nor exercise caused changes in the concentrations of GSH. In both groups had significantly higher concentrations of UA in both trials after 1 h of recovery than at rest and immediately post-exercise.

TAS values measured at rest and immediately post-exercise in the first trial were significantly different between the CU and the PL. In both groups, they were significantly higher after 1 h of recovery than immediately post-exercise. Significantly greater TAS concentrations (*p* ≤ 0.05) after 1 h of recovery than at rest were obtained for the PL in the first trial and for the CU in the second trial.

The resting levels of SIRT 3 obtained in the first trial were not significantly different between the groups. In the second trial, SIRT 3 levels significantly higher than at rest (*p* ≤ 0.01) were obtained in the PL group immediately after exercise, and after 1 h of recovery (Table 5). The CU group’ resting levels of SIRT3 (*p* ≤ 0.05) measured in the second trial were significantly greater than in the first trial, as well as significantly higher compared with the PL group. The resting levels of SIRT3 in the PL group in the second trial were significantly lower (*p* ≤ 0.05) than in the first trial.

### 3.3. Oxidative status

Supplementation with curcumin did not cause significant changes in the concentrations of oxidative stress markers (Table 3). In both groups and trials, the concentrations of TOS/TOC recorded immediately post-exercise were significantly higher than after 1 h of recovery. All participants had significantly higher plasma levels of MDA (*p* ≤ 0.05) in the second trial than in the first trial, but only in the PL group MDA levels measured immediately after exercise and 1 h later were significantly higher than before exercise (*p* ≤ 0.05). The CU group’s serum concentrations of 8-OHdG measured immediately after exercise were significantly lower (*p* ≤ 0.05) in the second trial than in the first trial; in the latter case, they were significantly higher (*p* ≤ 0.001) after 1 h of recovery than at rest.

**Table 3.** Changes in markers of oxidative stress after 6-week curcumin supplementation and exercise.

Variables	Measurement	Time	PL (n = 15)	CU (n = 15)
			Me (QD)	Me (QD)
TOS/TOC (µmol/l)	1-st	rest	295.1 (85.1)	322.6 (61.2)
		post test	376.6 (155.5)	405.3 (73.9)
		1h post test	284.3 (83.8) ^bbb^	291.0 (51.1) ^bbb^
	2-nd	rest	344.1 (155.1)	268.2 (121.3)
		post test	362.4 (196.1)	287.7 (171.5)
		1 h post test	267.3 (107.8) ^bbb^	245.2 (85.2) ^b^
MDA (µmol/l)	1-st	rest	4.8 (0.6)	4.8 (0.7)
		post test	4.2 (0.6)	4.7 (0.6)
		1 h post test	5.1 (0.9)	4.9 (0.5)
	2-nd	rest	5.5 (0.8) ^aa^	5.2 (0.5) ^a^
		post test	5.1 (0.9) ^aa^	4.7 (0.6)
		1 h post test	5.6 (0.6) ^aa^	5.2 (0.9)
8-OHdG (pg/ml)	1-st	rest	18640.0 (4296.0)	19600.0 (4812.0)
		post test	24418.2 (4513.5)	23812.4 (5276.5)
		1 h post test	27319.9 (7231.5)	25349.8 (6888.1)
	2-nd	rest	15048.0 (8020.0)	15368.0 (3772.0)
		post test	17737.2 (5502.8)	18474.8 (4300.2) ^a^
		1 h post test	22545.5 (5027.0)	22355.1 (4700.3) ^ccc^

Notes: Me – median; QD – quartile deviation; PL – placebo group; CU – curcumin – supplemented group; TOS/TOC – total oxidant status/total oxidant capacity; MDA – malondialdehyde; 8-OHdG-8-Hydroxy-2’-deoxyguanosine; ^a^*p *≤ 0.05, ^aa^*p *≤ 0.01 – significantly different *vs.* values in the first trial (Wilcoxon matched-pairs signed rank test); ^b^*p *≤ 0.05, ^bbb^*p *≤ 0.001 – significantly different *vs.* values immediately post-exercise in the same trial (Friedman rank test and Dunn’s post-hoc test); ^ccc^*p *≤ 0.001 – significantly different *vs.* values at rest in the same trial (Friedman rank test)

### 3.4. Markers of muscle damage

Curcumin supplementation did not have a significant effect on participants’ muscle damage markers (CK, LDH, and Mb) (Table 4). The PL group had a significantly higher activity of plasma CK after 1 h of recovery than at rest in both trials, and the CU group only in the first trial (*p* ≤ 0.05). In both trials, however, CK activity in the supplemented participants was significantly higher 1 h hour after exercise than immediately afterward.

**Table 4.** Changes in muscle-damage markers after 6-week curcumin supplementation and exercise.

Variables	Measurement	Time	PL (n = 15)	CU (n = 15)
			Me (QD)	Me (QD)
CK (U/l)	1-st	rest	180.8 (78.3)	126.8 (54.0)
		post test	197.2 (68.6)	136.9 (48.1)
		1h post test	212.7 (103.5) ^ccc^	151.4 (57.0) ^bbc^
	2-nd	rest	159.2 (112.0)	153.8 (85.1)
		post test	148.3 (123.1)	153.9 (84.2)
		1h post test	168.5 (153.6) ^c^	179.8 (94.7) ^bb^
LDH (U/l)	1-st	rest	269.8 (49.9)	291.4 (39.2)
		post test	309.8 (47.5)	331.7 (27.6)
		1h post test	303.5 (51.5) ^cc^	312.5 (27.7)
	2-nd	rest	327.1 (63.4) ^a^	299.5 (30.9)
		post test	386.2 (86.1)	327.3 (36.1)
		1h post test	349.3 (82.1)	324.8 (43.0) ^c^
Mb (ng/ml)	1-st	rest	8.3 (4.4)	8.6 (1.4)
		post test	8.5 (5.3)	10.4 (2.3)
		1h post test	15.8 (4.7) ^bbbccc^	13.5 (3.5) ^bbccc^
	2-nd	rest	8.7 (2.4)	8.4 (1.6)
		post test	10.9 (2.6)	9.4 (2.3)
		1h post test	13.6 (3.5) ^bbcc^	13.3 (3.6) ^bbcc^

Notes: Me – median; QD – quartile deviation; PL – placebo group; CU – curcumin – supplemented group; CK – creatine kinase; LDH – lactate dehydrogenase; Mb – myoglobin; ^a^*p *≤ 0.05 – significantly different vs. values in the first trial (Wilcoxon matched-pairs signed rank test); ^b^*p *≤ 0.05, ^bb^*p *≤ 0.01, ^bbb^*p *≤ 0.001 – significantly different *vs.* values immediately post-exercise in the same trial (Friedman rank test and Dunn’s post-hoc test); ^c^*p *≤ 0.05, ^cc^*p *≤ 0.01, ^ccc^*p *≤ 0.001 – significantly different *vs.* values at rest in the same trial (Friedman rank test)

Significantly higher activity of plasma LDH after 1 h of recovery than at rest was recorded for the PL group (*p* ≤ 0.01) in the first trial and for the CU group (*p* ≤ 0.05) in the second trial. In the PL group, LDH activity measured at rest in the second trial was significantly higher (*p* ≤ 0.05) than in the first trial.

The measurements of Mb concentrations showed that in both groups and trials, they were significantly higher (*p* ≤ 0.001) after 1 h of recovery than at rest and immediately post-exercise.

## 4. Discussion

The purpose of the study was to determine the effect of 6-week supplementation with a daily dose of 2 g of curcumin on blood prooxidant/antioxidant homeostasis, SIRT 3 level, and aerobic capacity of middle-aged amateur long-distance runners preparing for the fall competitions. Additionally, changes in the athletes’ prooxidant/antioxidant homeostasis induced by exercise tests conducted before and after curcumin supplementation were recorded and examined.

Regular physical training has a significantly different effect on the human body than a single workout session [34]. Unlike the latter, it brings about physiological adaptations to oxidative stress and improves the ability to cope with future challenges [35,36]. The adaptations improve the efficiency of enzymatic and non-enzymatic antioxidant defence systems and increase mitochondrial capacity to scavenge free radicals [37,38]. The ability of aerobic training to reduce the levels of oxidative stress markers is reported [39].

In contrast, a single session of high-intensity exercise offers limited adaptation to exercise associated with increased vasodilation and higher allosteric activity of enzymes, which may be insufficient to restore oxidant/antioxidant homeostasis [40]. According to research evidence, increased oxygen uptake during a long-distance run causes excessive production of ROS disturbing the runner’s prooxidant-antioxidant homeostasis [3,5,41–43].

Recently, researchers’ discussions have focused on the ability of supplementation with antioxidants such as polyphenols to eliminate or improve adaptive response to exercise. According to Gomez-Cabrera et al. [44], antioxidant supplements reducing the ROS levels may hinder beneficial cellular adaptations induced by physical exercise. However, other researchers indicate that polyphenols increase the antioxidant capacity of blood [45–47].

The content of methoxy, phenoxy, and carbon–carbon double bonds makes curcumin a potent antioxidant capable of directly and indirectly scavenging hydrogen peroxide, superoxide radicals, superoxide anion, hydroxyl radicals, singlet oxygen, nitric oxide, and peroxynitrite [48–51]. However, studies investigating the ability of curcumin supplementation to attenuate oxidative stress report inconclusive results, probably because poor intestinal absorption and rapid metabolism limit the bioavailability of curcumin. In order to increase it, curcumin supplements also include zinc, copper, magnesium, selenium ions, nanoparticles, liposomes, phospholipids or piperine [52,53].

Curcumin is reported to modulate the concentration of GSH and the activity of SOD and CAT enzymes that help neutralize free radicals and inhibit ROS-generating enzymes [54]. It is also indicated to activate the endogenous antioxidant defence mechanisms by modulating transcription factors [55], thereby influencing the levels of antioxidant enzymes, non-enzymatic antioxidants, and total antioxidant capacity (TAS). In our study, a daily dose of 2 g of curcumin that the participants took for a period of 6 weeks did not significantly improve their enzymatic and non-enzymatic antioxidant defences and did not have a significant effect on the concentration of UA. A slight (non-significant) increase in SOD activity was only recorded at week 6. Studies investigating the effect of curcumin supplementation (1.5 g/day for 28 days) on exercise-induced oxidative stress have not reported changes in participants’ total antioxidant capacity [19]. In our study, the activity of SOD and GPx and the concentration of GSH recorded after the exercise tests were not significantly different from those measured at rest. A significant increase in the concentration of plasma UA observed 1 h after exercise was consistent with other reports, according to which UA concentration takes some time to increase after strenuous exercise [43,56]. The significantly higher levels of serum TAS obtained 1 h after exercise in both supplemented and placebo groups were probably associated with increased plasma concentration of UA, which accounts for 50% of blood antioxidant capacity [56].

The effect of 6-week supplementation with curcumin on oxidative stress and oxidative damage was assessed by analysing TOS/TOC, 8-OHdG, MDA and Mb concentrations, and the activity of CK and LDH. Because curcumin is known to be able to permeate lipid membranes, change the mechanical properties of the bilayer and lipid domain behaviour [57], and reduce lipid peroxidation by oxidising Fe^3+^ free radicals [58], we expected that it would reduce the pro-oxidative effect of exercise on the study participants. However, only the concentrations of MDA changed (increased) significantly between baseline and week 6; the changes in the other oxidative stress markers (DNA damage and TOS/TOC) were not statistically significant. The activity of CK and LDH and the concentration of Mb did not change significantly, either. The activity of CK and LDH and the concentration of Mb recorded after the exercise tests were similar between the supplemented and placebo groups.

Because most ROS produced during endurance exercise come from the respiratory chain [59], we also evaluated the effect of curcumin on SIRT 3 levels. Even though they proved to be significantly higher after supplementation, a noticeable increase in SOD activity was not observed.

The measurements of maximal oxygen uptake, a widely used indicator of aerobic capacity, showed that its levels did not change in the participants who took a daily dose of 2 g of curcumin for a period of 6 weeks. However, some authors have presented evidence that curcumin influence the athletes’ VO_2max_ and improves their performance [60,61].

### 4.1. Study limitations

A limitation of the study was that the participants were not assessed for serum levels of curcumin and its metabolites.

## 5. Conclusions

The hypothesis that taking a daily dose of 2 g of curcumin for 6 weeks may enhance the antioxidant effect of physical training and improve the aerobic capacity of middle-aged amateur long-distance runners was not confirmed, because the levels of enzymatic and non-enzymatic antioxidants and oxidative stress markers did not change significantly in the supplemented group. More research is needed to ascertain whether supplementation with curcumin benefits endurance athletes during the preparatory phase of the macrocycle.

## Data Availability

The data used to support the findings of this study are available from the corresponding author upon request.
